# Ag Nanoparticles-Modified 3D Graphene Foam for Binder-Free Electrodes of Electrochemical Sensors

**DOI:** 10.3390/nano7020040

**Published:** 2017-02-16

**Authors:** Tao Han, Jianli Jin, Congxu Wang, Youyi Sun, Yinghe Zhang, Yaqing Liu

**Affiliations:** 1 Shanxi Province Key Laboratory of Functional Nanocomposites, North University of China, Taiyuan 030051, China; wuwg2010@163.com (T.H.); jianjl2017@126.com (J.J.); congxw2017@163.com (C.W.); yhe_zh@163.com (Y.Z.); 2 Nanotechnology Department, Helmholtz Association, Hamburg 21502, Germany

**Keywords:** in-situ approach, Ag nanoparticles, rGO foam, electrochemical detection, heavy metal ion

## Abstract

Ag nanoparticles-modified 3D graphene foam was synthesized through a one-step in-situ approach and then directly applied as the electrode of an electrochemical sensor. The composite foam electrode exhibited electrocatalytic activity towards Hg(II) oxidation with high limit of detection and sensitivity of 0.11 µM and 8.0 µA/µM, respectively. Moreover, the composite foam electrode for the sensor exhibited high cycling stability, long-term durability and reproducibility. These results were attributed to the unique porous structure of the composite foam electrode, which enabled the surface of Ag nanoparticles modified reduced graphene oxide (Ag NPs modified rGO) foam to become highly accessible to the metal ion and provided more void volume for the reaction with metal ion. This work not only proved that the composite foam has great potential application in heavy metal ions sensors, but also provided a facile method of gram scale synthesis 3D electrode materials based on rGO foam and other electrical active materials for various applications.

## 1. Introduction

Noble metal particles (e.g., Au, Pt and Ag) have attracted considerable attention due to their unique physical and chemical properties, as well as their potential use in electronic, photonic, catalytic and biological applications [1,2,3]. In particular, Ag nanoparticles are some of the well-developed materials that have been used for applications in electrochemical detection because they are inexpensive relative to other noble metal materials and they possess good chemical and physical properties [4,5,6,7,8,9,10,11,12,13,14]. For example, well-dispersed Ag nanoparticles have been successfully utilized in the electrochemical detection hydrogen peroxide [5,6,10,11,14], glucose [12], norepinephrine [13] and heavy metal ions including Hg(II) [7], Cr(IV) [8,9]. Unfortunately, in most cases, the Ag nanoparticles generated by the most methods were often in powder type, which were further coated on the glass carbon electrode (GCE) for electrochemical detection [4,5,6,7,8,9,10,11,12,13,14]. Therefore, the further electrode preparation led to additional cost and time. Furthermore, the low interaction and high contact resistance between Ag powder and glass carbon electrode would greatly decrease sensitivity and cycle stability of electrode, which greatly precluded their practical application in electrochemical detection. Therefore, there was still a need for further study to explore and develop the performance of Ag nanoparticles by effective routes in order to achieve high performance for electrochemical detection application.

Recently, reduced graphene oxide foam (rGO foam) has attracted a great deal of attention due to its low density, highly porous structures, extremely high electrical conductivity, large specific surface area, and so on [15,16]. As a result, the rGO foam was possible to serve as the working electrode replacing GCE for electrochemical detection application. Firstly, graphene foam not only acted as a working electrode, but also functions as an effective electrocatalyst. Moreover, the three-dimensional (3D) pore surface electrode based on graphene foam leaded to the enhanced electrocatalytic characteristics due to that the three dimensional architecture of rGO foam provided a large surface area, fast electron transport and allowed high accessibility to the reactants. Secondly, it was a convenient way of preparing freestanding electrode based on Ag nanoparticles for electrochemical detection by one step method, in which the Ag nanoparticles (Ag NPs) were in-situ grown on rGO foam. Moreover, the rGO foam could prevent the aggregation of Ag NPs, which provided its ability to create sensing system with fast electron transport, revisable, selective, and sensitive recognition over a wide range of concentrations and with the low detection limit in real-life samples [17]. In a word, the Ag NPs directly grown on rGO foam could act as a novel and excellent electrode for application in electrochemical detection sensor. However, there were few works reporting the Ag NPs/rGO composite foam electrode for application in electrochemical detection, especially, detection of heavy metal. In addition to this, Hg(II) was considered as one of the most dangerous metal ions for the environment and has most commonly toxic risks for human contacting areas as a result of natural processes [18,19]. The electrochemical detection methods were generally used for the detection of the Hg(II) due to their capability of short analytical time, low power cost, high sensitivity and easy adaptability for in-situ measurement [20,21,22]. However, there was still big challenge in terms of repeatability/reproducibility tests of electrochemical detection Hg(II). Therefore, it was critical to be able to detect and measure the level of Hg(II) in both environmental and biological samples under aqueous conditions with high repeatability/reproducibility.

Based on these facts, Ag NPs/rGO composite foam electrode has been fabricated simply through an in-situ method for a highly sensitive Hg(II) electrochemical sensor. Owing to the unique porous morphology, the Ag NPs/rGO composite foam electrode delivered significant electrochemical activity and cycling efficiency.

## 2. Experimental

### 2.1. Synthesis of Ag NPs/rGO Composite Foam

The Ag NPs/rGO composite foam was prepared by a facile method as shown in following. Firstly, graphene oxide (GO) was prepared from natural graphite powders by modified Hummers method, which has been reported in our previous work [23]. Secondly, the Ag NPs/rGO composite foam was prepared by the self-assembly method using a combination of L-ascorbic acid (L-AA) and HI as the reducing agents. In brief, 0.3 g L-AA and 0.3 mL HI were added to the 10.0 mL GO suspension (3.0 mg/mL) and AgNO_3_ (1.0 mg/mL). The mixture was sonicated for 10.0 min and then transferred to an oil bath at 95.0 °C for 6.0 h without stirring. The Ag NPs/rGO composite hydrogels was washed with water for 24.0 h to remove residual impurities and then the wet hydrogel was freeze dried for 12.0 h to obtain Ag NPs/rGO composite foam. In a comparison, the pure rGO foam was also prepared under the same conditions.

### 2.2. Characterization

X-Ray diffraction (XRD) (Bruker D8 ADVANCE, Billerica, MA, USA) was used to measure the crystal structure of the obtained samples, and the XRD patterns of samples (5°–80°) were measured.

The microstructure of composite foam was observed by scanning electron microscopy (SEM) (Su-8100, HITACHI, Tokyo, Japan) with an accelerating voltage of 20 kV.

Raman spectrum was collected on a Jobin-Yvon Lab Ram HR800 Raman spectroscope (JobinYvon, Paris, France) equipped with a 514.5 nm laser source.

N_2_ adsorption isotherms were characterized using Micromeritics Tristar BF-JW132 (Beijing JWGB SCI& Tech Co., Ltd, Beijing, China) and the specific surface area of the samples were calculated using Brunauer , Emmett and Teller (BET) method.

### 2.3. Electrochemical Characterization

All electrochemical measurements, including electrochemical impedance spectroscopy (EIS), cyclic voltammetry (CV) and differential square wave anodic stripping voltammetry (SWASV), were performed on a CHI1140A electrochemical workstation (CHI110, Austin, TX, USA). The Ag NPs/rGO composite foam electrode with 1.0 cm × 2.0 cm, Pt-wire and Ag/AgCl electrodes were used as working, counter and reference electrodes, respectively. All electrochemical measurements were carried out at room temperature. To eliminate the effect of dissolved oxygen, the electrolyte was purged with nitrogen gas for half an hour.

Hg(II) was analyzed by differential SWASV. A preconditioning of the Ag NPs/rGO composite foam electrode was carried out before each analysis by recording ten CV from −0.2 to +0.6 V at a scan rate of 100.0 mV·s^−1^ in electrolyte solution (0.1 M NH_3_). The Ag NPs/rGO composite foam electrode was immersed in 25.0 mL magnetically stirred standard solution or sample and Hg(II) were reduced to Hg(0) by applying a potential of −0.6 V for 180.0 s. Then, the electrode was rinsed with distilled water and transferred to another cell that contained of 25.0 mL 0.1 M NH_3_. Hg(II) analysis was carried out by SWASV from −0.65 to 0.55 V with 10.0 mV·s^−1^ scan rate, 25.0 mV modulation amplitude, 50.0 ms modulation time and 5.0 mV step potential.

## 3. Result and Discussion

**Figure 1:**
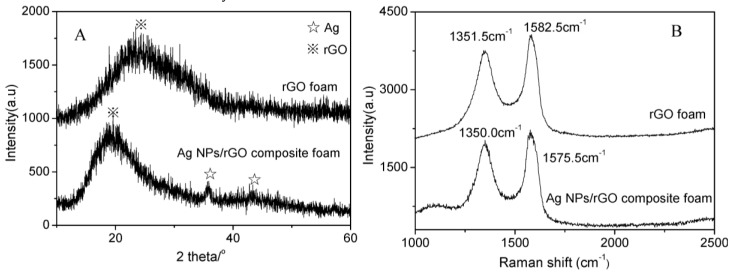
(**A**) X-Ray diffraction (XRD) and (**B**) Raman spectra of pure reduced graphene oxide (rGO) foam and Ag nanoparticles (Ag NPs)/rGO composite foam.

Figure 1A showed the XRD patterns of pure rGO foam and Ag NPs/rGO composite foam. It clearly showed one broad diffraction peak at around 22.0°, corresponding to the (002) plane of rGO for both samples [23]. At the same time, excluding the diffraction peak of rGO, there were other diffraction peaks located at 35.7° and 43.3°, which were ascribed to the (111) and (200) planes of Ag (JCPDS No. 04-0783), respectively [24]. The full width at half maximum (FWHM) value measured for (111) plane of reflection was used with the Debye-Scherrer equation to calculate the size of the Ag nanoparticles, and the average particle size was about 9.7 nm. In addition to this, the diffraction peak of rGO in Ag NPs/rGO composite foam was blue shift comparing to that of pure rGO foam. The result was attributed to increase of d-spacing between stack of graphene sheets due to introduce the Ag nanoparticles adsorbed on surface of rGO [25]. The Raman spectra of pure rGO foam and Ag NPs/rGO composite foam were shown in Figure 1B. The Raman spectrum of rGO foam showed two peaks at 1351.5 cm^−1^ and 1582.5 cm^−1^, corresponding to the D band and G band of rGO, respectively [26]. In comparison to rGO foam, the Raman spectrum of Ag NPs/rGO composite foam indicated that the D and G band shifted to 1350.0 cm^−1^ and 1575.5 cm^−1^, respectively. In addition, a new Raman peak at 1100.0 cm^−1^ was present, resulting from the interaction between rGO and Ag nanoparticles [27]_._ These results indicated the formation of Ag NPs/rGO composite foam, in which the Ag nanoparticles were anchored on rGO foam by the chemical interaction.

**Figure 2:**
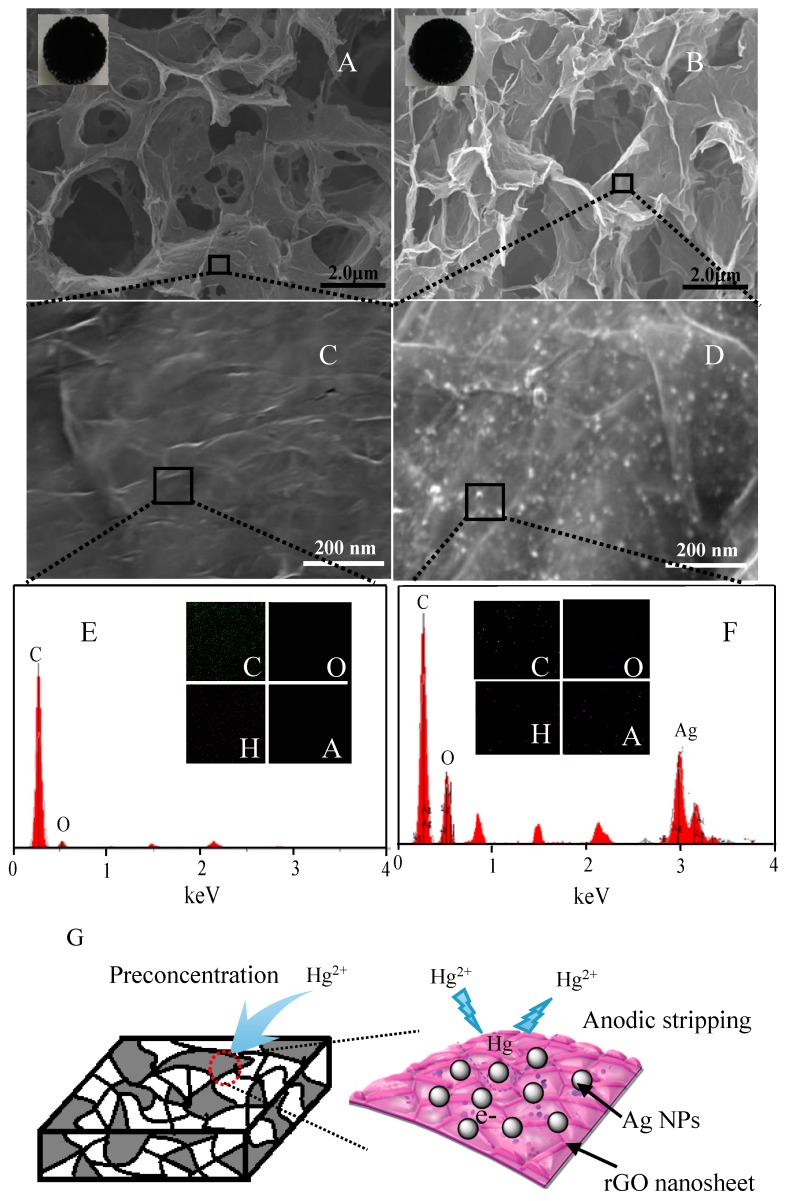
Low-magnification scanning electron microscopy (SEM) images of (**A**) rGO foam and (**B**) Ag NPs/rGO composite foam; (**C**) and (**D**) are the enlarged view of the black square dashed box marked in (**A**,**B**), respectively. The inset of (**A**,**B**) are digital photographs of foams. Energy dispersive spectrometer (EDS) spectra of (**E**) rGO foam and (**F**) Ag NPs/rGO composite foam. The inset of (**E**,**F**) are elemental mapping image of rGO foam and Ag NPs/rGO composite foam, respectively; (**G**) is the schematic diagram of the Ag NPs/rGO composite foam structure.

The morphologies of pure rGO foam and Ag NPs/rGO composite foam were investigated by the SEM as shown in Figure 2. Obviously, it could be seen that the rGO foam and Ag NPs/rGO composite foam both exhibited 3D network with highly open and porous structure as shown in Figure 2A,B, respectively. The insets of Figure 2A,B showed the optical images of the pure rGO foam and Ag NPs/rGO composite foam. The surface color of the both foams was clearly black, demonstrating the formation of rGO foam and Ag NPs/rGO composite foam. By a closer examination of the pure rGO foam and Ag NPs/rGO composite foam, it was found that the nanoparticles were well dispersed on surface of pore wall as shown in Figure 2D. In addition, there was not large aggregate on surface of pore wall. Moreover, this was further characterized by the energy dispersive spectrometer (EDS) spectrum and elemental mapping image as shown in Figure 2F. It clearly presented the C and Ag element for the composite foam. Contrarily, there were few particles in surface of pore wall and the Ag element was not observed, as shown in Figure 2C,E. These results further indicated the formation of Ag NPs/rGO composite foam, in which the Ag nanoparticles were well dispersed in composite foam. The 3D network of rGO foam and good distribution of Ag NPs revealed the existence of more channels and active surface area within the composite foam, which allowed metal ions easily penetrate to the inner of composite and thus accelerated the interfacial reaction. In addition, the Ag NPs was anchored on rGO foam by the chemical interaction, which reduced contact resistance and improved the cycle stability of electrode. So, the Ag NPs/rGO composite foam was suited to applications in electrochemical detection as shown in Figure 2G.

**Figure 3:**
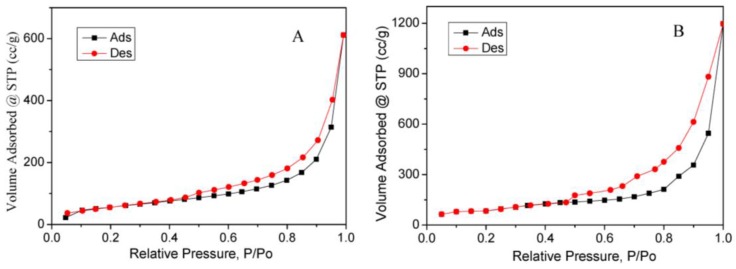
Nitrogen adsorption-desorption isotherm curve of (**A**) pure rGO foam and (**B**) AgNPs/rGO composite foam.

Figure 3 shows the N_2_ adsorption-desorption isotherms of the pure rGO foam and Ag NPs/rGO composite foam. All of the curves exhibited type IV isotherms with hysteresis loops of different sizes, which reflected the propensity of mesoporous material to facilitate electrochemical reaction during the electrochemical detection process [28]. The BET surface area of the pure rGO foam and Ag NPs/rGO composite foam was calculated to be about 229.3 m^2^·g^−1^ and 327.4 m^2^·g^−1^, respectively. The result was attributed to the synergistic effect of Ag NPs and rGO. The rGO could effectively prevent the aggregate of Ag NPs. At the same time, the Ag NPs also could prevent the stack of rGO. So, the Ag NPs/rGO composite foam could offer a larger BET surface area. It was well-known that the surface area was an important parameter for any material to deliver excellent electrochemical performances. The large surface area leaded to more active sites to be accessed by metal ion during the electrochemical reaction [28].

**Figure 4:**
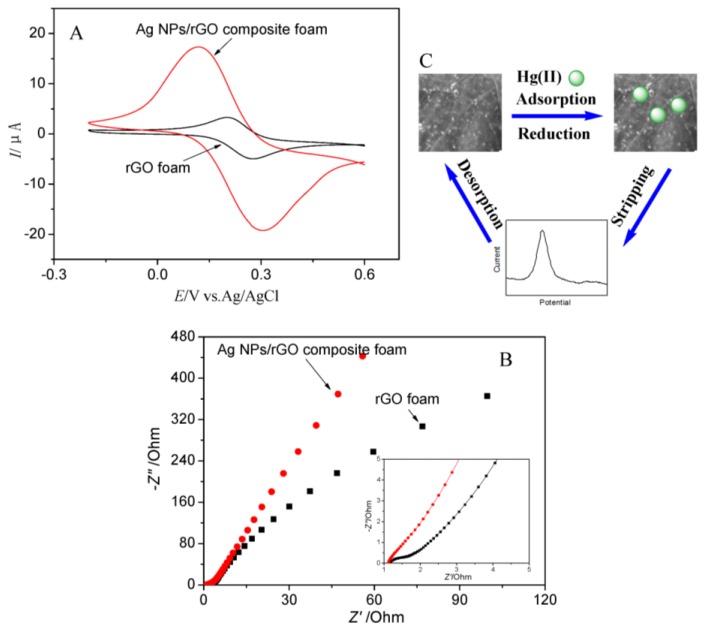
(**A**) Cyclic voltammetry (CV) and (**B**) electrochemical impedance spectroscopy (EIS) of pure rGO foam and Ag NPs/rGO composite foam (EIS parameters: potential, 0.21 V; frequency range, 100 kHz–0.1 Hz; amplitude, 5.0 mV); (**C**) Schematic representation of electrochemical detection toward Hg(II) by Ag NPs/rGO composite foam.

The pure rGO foam and Ag NPs/rGO composite foam were characterized using CV and EIS as shown in Figure 4A,B, respectively. Compared with the Ag NPs/rGO composite foam, the pure rGO foam exhibited the lower intensity current peaks due to the quasi-super hydrophobicity which was attributed to poor surface wetting leading to a reduced access to and limited utilization of the available surface area of pristine graphene [29]. In addition to this, the shifting of the cathodic (reduction reaction) and anodic (oxidation reaction) peaks may be due to the better interaction between Ag NPs and Hg(II) and more active sites in the Ag NPs/rGO composite foam electrodes [30,31]. These results indicated that the Ag NPs/rGO composite foam electrode had better electrochemical catalytic behavior and promotion of electron transfer process as compared to pure rGO foam [32]. The interface properties of the pure rGO foam and Ag NPs/rGO composite foam electrode were further investigated using EIS as shown in Figure 4B. The Nyquist plots include a semicircle portion at higher frequencies corresponding to the electron-transfer-limited process and a linear part at lower frequency range representing the diffusion-limited process. Charge transfer resistance (*R_ct_*) of the electrode can be obtained from the diameter of the semicircle. The curves of Ag NPs/rGO composite foam electrode exhibited a gradually smaller radius of semicircles compared with the pure rGO foam electrode. The results indicated that the Ag NPs/rGO composite foam could enhance electron-transfer between the electrode and the electrochemical probe, which was very important to improve the sensitivity of electrochemical detection [33]. A schematic drawing of Hg(II) detection mechanism for the Ag NPs/rGO composite foam electrode was shown in Figure 4C. In the progress of detection, the Hg(II) were adsorption by surface precipitation of metal hydroxide/carbonate onto the surface of Ag NPs/rGO , then reduced (Hg(II) to Hg(0) ) and deposited on the surface of electrode at a certain potential. At the step of anodic stripping, the electrodeposited metals Hg(0) were reoxidized to metal ions Hg(II) with the appearance of stripping peak in a potential range for the identification. Finally, the residual Hg on the electrode surface was removed at a positive potential. The electrochemical responses could be enhanced, and this was attributed to the synergistic and amplifying effects of Ag NPs/rGO composite foam.

**Figure 5:**
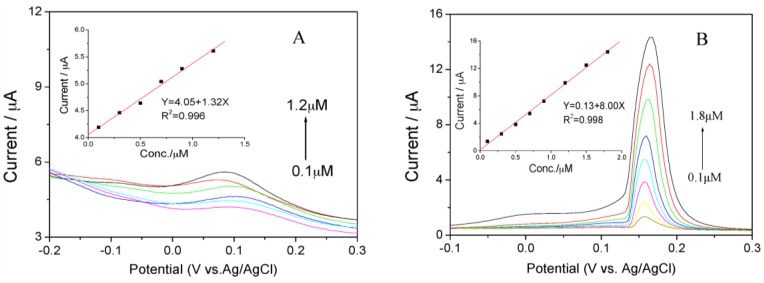
Square wave stripping voltammograms (SWASV) responses of (**A**) pure rGO foam and (**B**) Ag NPs/rGO composite foam electrode towards Hg(II) at different concentrations in 0.1 M NH_3_ solution. The insets correspond to the calibration plots of foam electrodes.

Under the optimal experimental conditions, Hg(II) was determined on pure rGO foam and Ag NPs/rGO composite foam electrode using SWASV as shown in Figure 5. Figure 5A showed the SWASV responses of the pure rGO foam toward Hg(II) over the concentration range of 0.1–1.2 µM in 0.1 M NH_3_. The limit of detection (LOD) was calculated to be 0.12 µM (3σ method) with a sensitivity of ca. 1.32 µA/µM. Similarly, Figure 5B presented the SWASV responses of the Ag NPs/rGO composite foam electrode toward Hg(II) over the concentration range of 0.1–1.8 μM in 0.1 M NH_3_. As seen from the calibration plot of Hg(II) in inset of Figure 5B, the peak currents increased linearly versus the Hg(II) concentrations with the sensitivity of ca. 8.0 µA/µM, and the LOD was calculated to be 0.11 µM (3σ method). It could be clearly observed that the sensitivity and LOD of Ag NPs/rGO composite foam for analysis of Hg(II) was better compared with the results of pure rGO foam. The sensitivity and LOD of the present study, together with previously determined values for electrochemical sensing in various other electrodes based on Ag nanoaprticles/rGO composite powders, were also summarized in Table 1. It could be observed that the preferable sensitivity and LOD could be obtained at the Ag NPs/rGO composite foam electrode. Then the results demonstrated that the Ag NPs/rGO composite foam could be a free-standing electrochemical electrode applied in electrochemical detection of heavy metal ions. The high sensitivity and LOD was attributed to the following two reasons: (1) the integration of the working electrode (rGO foam) and the electrocatalyst (Ag) eliminated the hetero-interface and the contact resistance between them, leading to fast electron transport; (2) the Ag grown on the surface of rGO nanosheets in rGO foam had a 3D network structure, which could offer a large active surface area and more active sites to be accessed by metal ion, and enables rapid ion transport.

**Figure 6:**
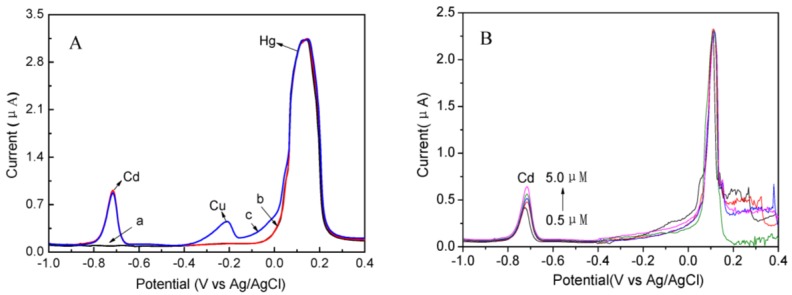
(**A**) Interference studies of typical SWASV responses of 0.4 μM Hg(II) on Ag NPs/rGO composite foam electrode in 0.1 M NH_3_ (**a**) without other metal ion, (**b**) in the presence of Cd(II), (**c**) in the presence of Cd(II) and Cu(II); (**B**) Interference studies of typical SWASV responses of 0.3 μM Hg(II) on Ag NPs/rGO composite foam electrode in 0.1 M NH_3_ in the presence of Cd(II) with various concentration.

In order to investigate the interference of the other heavy metal ions to electrochemical detection of Hg(II), a series of interference measurements were studied as shown in Figure 6. Figure 6A showed the SWASV responses of the Ag NPs/rGO composite foam electrode toward Hg(II) (0.4 μM) in 0.1 M NH_3_ with the existing of other heavy metal ions (Cd(II) and Cu(II)). The peak current of Hg(II) was almost unchanged in the presence of 1.0 μM Cd(II) and Cu(II). In addition to this, the peak current of Hg(II) was also almost similar in the presence of Cd(II) with various concentrations. The possible reason for this was the unobvious interference of Cd(II) and Cu(II) on the adsorption ability of Ag NPs/rGO composite foam toward Hg(II). The result indicated that the Hg(II) could be detected in complex samples with minimum interference from other heavy metal ions. In addition, it also indicated that the Ag NPs/rGO composite foam as binder-free electrodes could be applied in electrochemical detection of Hg(II) in real waste water.

**Figure 7:**
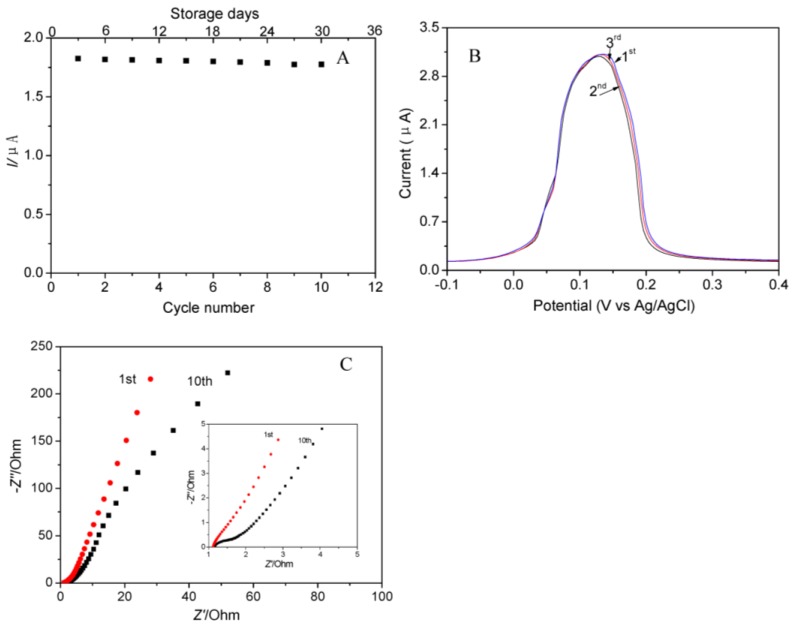
(**A**) SWASV responses of Ag NPs/rGO composite foam electrode towards Hg(II) from 1st cycle to 10th cycle; (**B**) SWASV responses of three Ag NPs/rGO composite foam electrode prepared at the same procedure; (**C**) EIS of Ag NPs/rGO composite foam electrode towards Hg(II) from 1st cycle to 10th cycle.

The stability of the electrode was very important for practical application in electrochemical detection. The cycling performance of the Ag NPs/rGO composite foam electrode towards detection of Hg(II) at 0.11 µM after various cycles of storage in air for 3 days and the peak current was shown in Figure 7A. The peak current slightly decreased with an increase in cycling test and become stable after 10 cycling tests. The relative standard deviation (RSD) of peak current was only 0.46% for the 10th cycling test and storage in air for 30 days, which was far lower than the RSD of the peak current reported in previous works as shown in Table 1. The result confirmed that the Ag NPs/rGO composite foam binder-free electrode exhibited better cycling stability and long-term durability as a sensor of Hg(II) compared with the Ag NPs modified GCE electrode. This result was attributed to the following two reasons: (1) there was stronger interaction between the working electrode (rGO foam) and the electrocatalyst (Ag) comparing to GCE and active materials; (2) the Ag NPs grown on the surface of rGO nanosheets in rGO foam had a 3D network structure, which could reduce volume expansion/shrinkage. Furthermore, the reproduction stability of the Ag NPs/rGO composite foam electrode towards detection of Hg(II) at 0.4 µM was shown in Figure 7B. It clearly showed that the peak current demonstrated a slight change for the three Ag NPs/rGO composite foam electrodes prepared with the same procedure. The RSD value of the peak current was about 0.58%. The result indicated that the Ag NPs/rGO composite foam electrode also exhibited reproduction stability as a sensor of Hg(II). In order to study the kinetics and mechanism of high cycling stability, the EIS of Ag the NPs/rGO composite foam electrode before and after 10 cycling test was compared as shown in Figure 7C. It clearly showed that there was almost no change in ESR before and after 10 cycling tests. The result indicated that the effect of cycling test on the internal resistance of the Ag NPs/rGO composite foam was slight.

## 4. Conclusions

Ag NPs directly grown on rGO foam were successfully synthesized by a one-step in-situ approach. The Ag NPs/rGO composite foam electrode offered excellent electrocatalytic ability toward Hg(II). In the amperometric detection of Hg(II), the line arrangement, sensitivity, and limit of detection were 0.1–1.8 µM (correlation coefficient, *R*^2^ = 0.997), 8.0 µA/µM, and 0.11 µM, respectively. The electrocatalytic current of the Ag NPs/rGO foam electrode toward Hg(II) still remained 97.5% and became stable after 30 days of storage in atmosphere. The relative standard deviation of current is only 0.46% for 10 cycling tests, indicating the excellent electrode cycling stability and long-term durability. These findings showed the applicability of the Ag NPs/rGO foam electrode as a reliable Hg(II) sensor.

## Figures and Tables

**Table 1:** Comparison of current sensitivity and limit of detection (LOD) with previously reported values of different electrodes.

Electrodes	Sensitivity (µA/µM)	LOD (µM)	Detection Materials	References
Pure reduced grpahene oxide (rGO) foam	1.32	0.12	Hg(II)	This work
Ag NPs/rGO composite foam	8.00	0.11	Hg(II)	This work
Ag NPs/rGO/Ni composite foam	1.09	14.90	H_2_O_2_	[24]
N-rGO/AgNPs/GCE	3.12 × 10^−3^	1.20	H_2_O_2_	[34]
AgNPs/PD-rGO/GCE	0.01	2.07	H_2_O_2_	[35]
PDDA-rGO/AgNPs/GCE	9.17 × 10^−3^	35.00	H_2_O_2_	[36]
